# Tumor budding in preoperative breast biopsies predicts sentinel lymph node metastasis

**DOI:** 10.17305/bb.2025.13323

**Published:** 2025-11-07

**Authors:** Songul Peltek Ozer, Bahri Ozer, Gulali Aktas

**Affiliations:** 1 Department of Pathology, Abant Izzet Baysal University Hospital, Bolu, Türkiye; 2 Department of General Surgery, Abant Izzet Baysal University Hospital, Bolu, Türkiye; 3 Department of Internal Medicine, Abant Izzet Baysal University Hospital, Bolu, Türkiye

**Keywords:** Breast cancer, tumor budding, sentinel lymph node biopsy

## Abstract

Sentinel lymph node biopsy (SLNB) is a pivotal technique employed to assess the necessity for axillary lymph node dissection (ALND), evaluated during the preoperative phase through clinical and radiological findings. The preoperative identification of sentinel lymph node metastasis has gained paramount importance in the surgical management of breast cancer. Tumor budding (TB) has emerged as a significant prognostic marker across various cancers, including breast cancer, where it is instrumental in detecting lymph node metastasis. This study aims to investigate the role of TB in predicting sentinel lymph node metastasis in preoperative breast biopsies. We included patients diagnosed with breast cancer, specifically those with invasive ductal carcinoma (IDC), who underwent preoperative needle biopsy and subsequent evaluation of postoperative surgical specimens, as well as SLNB at our medical center. The histological slides of these cases were reevaluated, and tumor cell clusters comprising up to four cells were classified as TB. Lymph nodes exhibiting tumor cell involvement, limited to macrometastasis, were classified as positive. A total of 65 patients were enrolled in the study. Among these, 36 patients exhibited TB in their preoperative biopsies, while 29 did not. The median tumor sizes were 20 mm (range: 6–50 mm) in the TB-positive group and 19 mm (range: 2–50 mm) in the TB-negative group (*P* ═ 0.3). Sentinel lymph node metastasis was detected in 18 patients with TB, compared to only five patients without TB, a difference that was statistically significant (*P* ═ 0.006). We conclude that evaluating TB in breast tru-cut specimens, in conjunction with clinical and radiological findings, may enhance the preoperative assessment of breast cancer cases requiring SLNB.

## Introduction

Breast cancer remains the most frequently diagnosed cancer among women, with axillary lymph node metastasis serving as a critical prognostic factor in early-stage breast cancer. Since William Halsted performed the first radical mastectomy in 1882—entailing the removal of all breast tissue, axillary lymph nodes, and both pectoralis muscles—minimally invasive treatment approaches have evolved significantly [1]. Despite advancements, axillary lymph node dissection (ALND) has long been essential for staging and local-regional control, though the decision to perform ALND must be carefully considered due to its associated complications.

Sentinel lymph node biopsy (SLNB), a surgical technique complemented by pathological assessment, aids in guiding the decision-making process for ALND, alongside clinical and radiological evaluations conducted preoperatively. ALND has consistently played a significant role in ensuring local-regional control and accurate staging of breast cancer [2].

The selection process for patients requiring ALND has become increasingly rigorous due to potential postoperative complications, such as mobility impairment, paresthesia, lymphedema, seroma, and pain, which can adversely affect the quality of life in approximately 80% of patients. Notably, axillary lymph node metastasis goes undetected in around 80% of early-stage breast cancer cases [1, 3, 4]. Advanced imaging techniques, including ultrasonography and PET/CT, as well as physical examination of the axilla through palpation, lack sufficient sensitivity, particularly in cases of micro-metastasis, underscoring the importance of SLNB [3, 5].

Tumor budding (TB), first described in colorectal cancer and defined as the presence of single malignant cells or clusters of fewer than five malignant cells, was standardized at the Tumor Budding Consensus Conference [6, 7]. Research has increasingly linked TB to epithelial–mesenchymal transition (EMT), as observed in various organ tumors. Studies have identified TB as a novel prognostic marker, independent of tumor stage and grade, in esophageal, gastric, and pancreatic cancers [7, 8].

In breast cancer, TB has emerged as a significant indicator of lymph node metastasis and correlates with poor prognoses and reduced survival rates [9]. The present study aims to evaluate the role of TB in preoperative breast biopsies as a predictor of sentinel lymph node metastasis.

## Materials and methods

This study included cases diagnosed with breast cancer at Bolu Izzet Baysal University Hospital between 2018 and 2023, specifically those with invasive ductal carcinoma (IDC) as the histological type. All cases underwent preoperative needle biopsy and subsequent SLNB at our center. Exclusions were made for cases with other histological types, those whose biopsies were conducted at different medical centers, patients who underwent ALND without SLNB, and those who received neoadjuvant therapy. The slides from the cases were re-evaluated.

TB, standardized through a consensus-based evaluation system, is characterized by isolated tumor cells separated from the main tumor mass or clusters of up to four cells [10]. Due to the inability to determine whether the biopsy material represented peripheral or central tumor areas, TB was classified without distinction as intra- or peritumoral. Following consensus recommendations, the area with the highest TB density was selected, and the number of buds was counted within an area of 0.785 mm^2^, without performing routine immunohistochemical examinations. All tumor areas in the specimens were evaluated.

**Figure 1.**
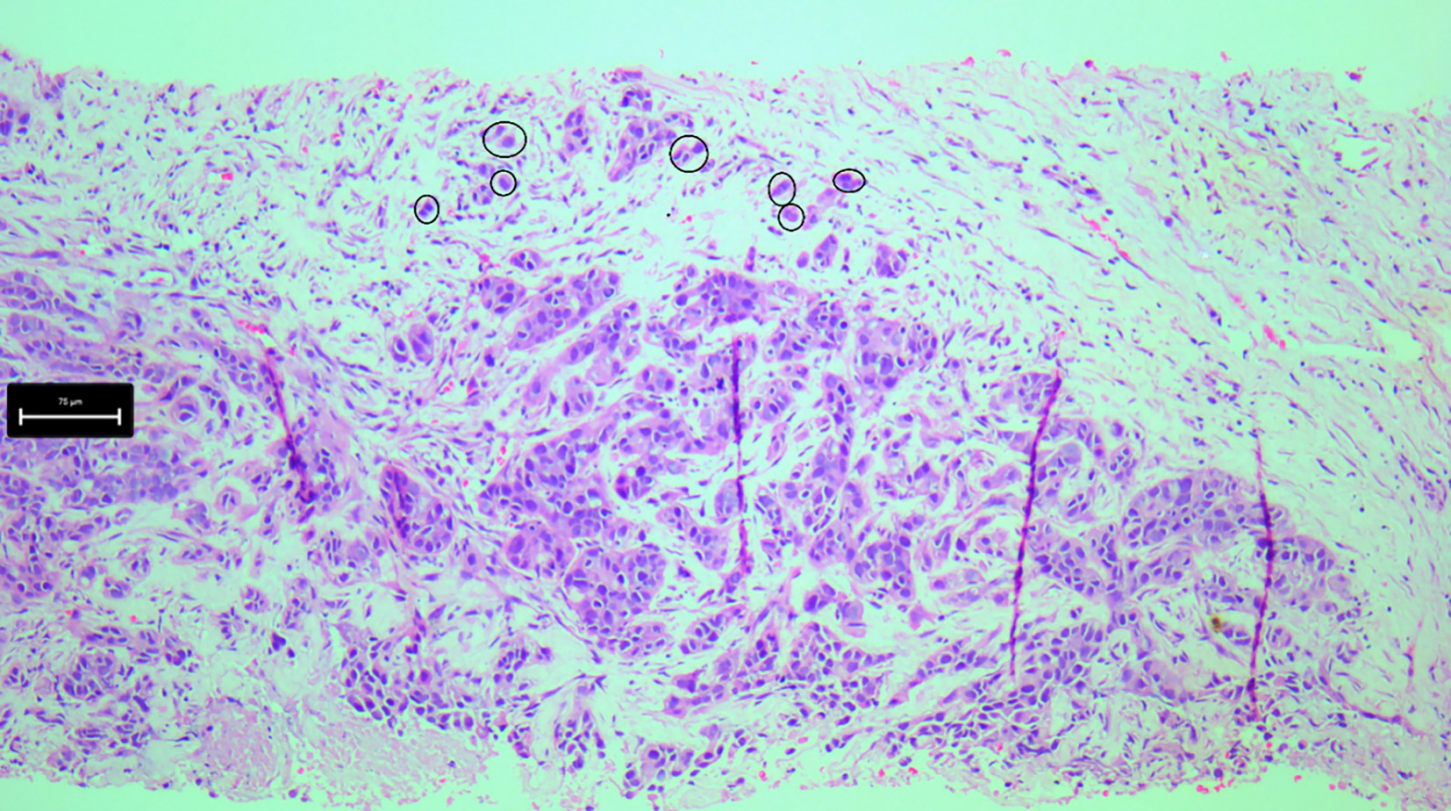
**Tumor budding (TB) detached from the main tumor mass in biopsy specimens.** Tumor cell clusters of up to four cells were categorized as TB, with black circles denoting tumor buds (H&E stain, ×100).

In biopsy specimens, tumor cell clusters of up to four cells were classified as TB (Figure 1). A single pathologist evaluated all tumor areas in the specimens without blinding. The cases were subsequently categorized into two groups: those with TB and those without TB.

Sentinel lymph node dissection was performed via the axilla following methylene blue injection into the nipple–areolar complex, targeting a total of three lymph nodes, at least two of which were stained. The median excised sentinel lymph node counts were as follows: in the TB present group, a median of 3 (range: 3–4); and in the TB absent group, a median of 3 (range: 3–4). The excised lymph nodes were examined in serial sections, with the study including only cases exhibiting macrometastasis, and immunohistochemical examination was not performed.

A total of 82 subjects were initially enrolled, after exclusion of 17 according to the exclusion criteria, data of remaining 65 subjects were enrolled to the study.

### Ethical statement

Ethical approval was obtained from the institutional ethics committee at Abant Izzet Baysal University (approval date: October 25, 2023; approval number: 2023/345). The study adhered strictly to the principles outlined in the Declaration of Helsinki.

### Statistical analysis

Statistical analyses were performed using IBM SPSS version 20.0 software. Normality was assessed using the Shapiro–Wilk test. Variables conforming to a normal distribution were compared using an independent samples *t*-test and are presented as means (±SD). Non-normally distributed continuous variables were analyzed using the Mann–Whitney *U* test and are expressed as medians (min–max). Categorical variables were compared using the chi-square test and are reported as counts and percentages. Receiver operating characteristic (ROC) curve analysis was employed to determine the sensitivity and specificity of TB in detecting sentinel lymph node positivity. The Youden index was utilized to establish cutoff values in the ROC analysis. A binary logistic regression analysis, accounting for patient age, tumor size, multicentricity, and TB, was conducted to assess whether TB serves as an independent risk factor for sentinel lymph node metastasis. The Hosmer–Lemeshow calibration method was applied during the binary logistic regression analysis. A *P* value of <0.05 was considered statistically significant for all analyses.

## Results

Among the patients, 36 (55.4%) exhibited TB in their preoperative biopsies, while 29 (44.6%) did not. The mean ages of the TB-positive and TB-negative groups were 53 ± 12 years and 54 ± 13 years, respectively (*P* ═ 0.77). The average tumor size for patients with TB was 20 mm (range: 6–50 mm), while it was 19 mm (range: 2–50 mm) for patients without TB (*P* ═ 0.3). Multicentricity was identified in 4 (11%) patients with TB and in 2 (7%) patients without TB (*P* ═ 0.56). Sentinel lymph node positivity was observed in 18 (50%) patients with TB, compared to 5 (17%) patients without TB. This difference was statistically significant (*P* ═ 0.006) (Table 1).

**Table 1:** Characteristics of the study population

		**TB present**	**TB absent**	***P***
		Mean ± SD*		
Age (years)		53 ± 12	54 ± 13	0.77
		Median (min.–max.)**		
TB count (*n*)		11 (2–50)	0 (0-0)	**<0.001**
Size (mm)		20 (6–50)	19 (2–50)	0.3
		*n,* (%)***		
Multicentricity	Positive (*n*, (%))	4 (11)	2 (7)	0.56
	Negative (*n*, (%))	32 (89)	27 (93)	
Sentinel LN	Positive (*n*, (%))	18 (50)	5 (17)	**0.006**
	Negative (*n*, (%))	18 (50)	24 (83)	

*Independent samples *t*-test; **Mann–Whitney *U* test; ***chi-square test. Abbreviations: TB: Tumor budding; SD: Standard deviation; LN: Lymph node.

In the ROC analysis, a TB count exceeding 3 demonstrated a sensitivity of 73% and a specificity of 62% in detecting sentinel lymph node positivity (AUC: 0.69, *P* ═ 0.01, 95% CI: 0.55–0.82). The positive and negative predictive values of TB for detecting sentinel lymph node metastasis were 72% and 57%, respectively. The binary logistic regression analysis revealed that the absence of TB independently reduced the likelihood of sentinel lymph node metastasis by 80% (*P* ═ 0.013, OR = 0.20, 95% CI = 0.06–0.71) (Table 2).

**Table 2:** Results of regression analysis

	***P***	**OR**	**95% CI**
Age	0.68	1.01	0.963–1.059
Tumor size	0.33	0.98	0.926–1.026
Multicentricity	0.054	0.1	0.009–1.039
Tumor budding	0.01	0.2	0.056–0.712

Abbreviations: OR: Odds ratio; CI: Confidence interval.

## Discussion

The present study demonstrated that sentinel lymph node positivity was more prevalent in patients with TB compared to those without. Additionally, the findings suggest that TB in preoperative biopsies is not associated with age, tumor size, or multicentricity, but is significantly correlated with an increased rate of sentinel node positivity. This indicates that patients exhibiting TB in their biopsies are more likely to have nodal metastasis. Consequently, TB may serve as a predictor of a more aggressive cancer phenotype, regardless of tumor size and other factors in breast cancer patients. These results support the hypothesis that TB is a morphological marker of early invasion and partial EMT, as well as early metastatic potential.

The concept of TB has been better studied in colorectal cancer, but in recent years there’s growing interest in breast cancer. A key study by Salhia et al. [11] found that high peripheral TB in surgical specimens was associated with lymph node metastases and lymphatic invasion. In the matched preoperative biopsies in that study, high TB was associated with venous invasion [11]. Thus, our finding (of higher SLN positivity in TB-positive biopsies) is consistent with that prior association.

More broadly, Gujam et al. [12] observed TB in a larger cohort of ductal breast cancers and found that higher TB was associated with adverse pathologic features including lymph node involvement, lymphovascular invasion, and worse cancer-specific survival, independent of other factors. Similarly, in 2023, Ozer [13] also studied TB in invasive ductal breast carcinoma, examined correlations with clinicopathologic parameters and reported that higher TB was often correlated with adverse features. More recently, Ranaee et al. [14] revealed a significant relationship between the number of tumor buds (both intratumoral and peritumoral) and tumor size, stage, number of involved lymph nodes, and 5-year survival, though they did not find a significant association with age or tumor grade. That is quite consistent with our observations, lack of association with age, but correlation with nodal involvement.

A 2025 study by Shah et al. [15] similarly confirmed that high TB was associated with poor prognostic features, including higher tumor grade, negative hormone receptor status, and advanced T stage. The results of the present study are consistent with existing literature, reinforcing TB as a morphological predictor of nodal metastasis and worse outcomes, independent of tumor size, grade, or patient age. One distinguishing aspect of our study is that, while many prior investigations assessed TB in surgical specimens post-resection, we analyzed preoperative biopsies. This distinction is critical; if TB can be reliably evaluated in biopsies and predicts nodal metastasis, it carries significant clinical implications for preoperative risk stratification, impacting decisions regarding axillary surgery and neoadjuvant therapy. Salhia et al.’s work has already explored TB in core biopsies vs resections, demonstrating that high TB in biopsy specimens significantly correlates with venous invasion, thereby supporting the relevance of budding in biopsy analysis [11]. Our findings extend this knowledge by linking biopsy TB to sentinel node positivity.

In a recent systematic review and meta-analysis by Buch et al. [16], the authors identified high-grade TB as an independent risk factor for lymph node metastasis and lymphovascular invasion. Another meta-analysis examining TB in oral squamous cell carcinoma found that while TB was a significant predictor of overall survival, it did not correlate with lymph node metastasis [17]. Additionally, large observational studies consistently indicate that elevated TB counts are associated with greater lymph node involvement and other adverse features [11, 12, 18]. Our findings align with the existing literature.

TB is acknowledged as a morphological marker indicative of early invasion and EMT. It is consistently associated with a heightened probability of nodal metastasis and poorer outcomes across multiple studies and meta-analyses. These findings support TB’s potential role as a preoperative risk marker [19, 20]. However, the literature highlights the necessity for standardization in TB scoring—encompassing definitions, field sizes, and cutoffs—as well as the need for larger, validated cohorts before routine clinical implementation. Buch et al. [16] specifically noted the heterogeneity in TB assessment and the imperative for standardized reporting. Furthermore, the behavior of TB may vary among molecular subtypes (ER/PR/HER2), with some studies demonstrating differing associations based on subtype. For instance, Gujam et al. [12] established that TB’s prognostic value was independent of molecular subtype. However, due to the small sample size, our study could not perform subgroup analyses.

Our study demonstrated that the absence of TB reduced the risk of sentinel lymph node metastasis by 80%, independent of age, tumor size, and multicentricity. This indicates that, after adjusting for other covariates, patients lacking TB had one-fifth the odds of sentinel lymph node metastasis compared to those with TB. According to our analysis, TB remained a robust independent predictor of nodal metastasis, even when considering tumor size, multicentricity, and age. This observation is consistent with previous research in breast cancer, which has shown a correlation between elevated TB and lymphatic invasion as well as nodal metastasis [11]. Larger cohorts also reveal an independent association between TB and adverse outcomes, including nodal disease and diminished cancer-specific survival [12]. Meta-analytic data further corroborate this relationship, indicating that high TB approximately doubles the odds of lymph node metastasis (pooled odds ratio of around 2.25), while also being associated with lymphovascular invasion across studies [16]. Our adjusted effect (inverse of odds ratio: 0.20, corresponding to an odds ratio of 5 for the presence of TB) exceeds pooled estimates, yet remains biologically plausible. TB likely reflects EMT and an invasive cellular phenotype that is not fully captured by tumor size or multicentricity alone. Given the heterogeneity in TB assessment and the modest sample size in our study, these findings necessitate validation in larger, standardized cohorts, as well as the inclusion of additional covariates (e.g., lymphovascular invasion, grade, and molecular subtype) to confirm the independent prognostic utility of TB in preoperative decision-making.

The rising incidence of breast cancer, coupled with the increasing adoption of minimally invasive approaches and the complications associated with ALND, underscores the critical importance of SLNB, particularly in early-stage patients [5, 9]. Despite advances in physical examination and radiological imaging, the selection of patients for SLNB during the preoperative period has become increasingly significant.

The primary scenarios in which SLNB is applicable include: patients with clinically negative axillary lymph nodes, patients with clinically negative axillary lymph nodes even in cases of multicentric disease, patients planned for breast-conserving surgery and radiotherapy, and patients undergoing neoadjuvant therapy who present with clinically negative axillary lymph nodes prior to treatment, or those who have clinically positive axillary lymph nodes before treatment that become negative post-treatment [21, 22].

ALND remains a significant contributor to postoperative complications in breast cancer patients. Consequently, the decision to perform ALND is now approached with greater caution, with efforts to avoid it whenever possible. While clinical examination and radiological imaging play key roles in determining the necessity of ALND, these methods are often inadequate, particularly in the presence of micrometastasis, wherein SLNB becomes essential [3].

The primary situations in which axillary dissection is not recommended, even in the presence of micrometastases in SLNB, are as follows: patients with a tumor diameter of ≤5 cm, patients with micrometastases in 1–2 sentinel lymph nodes, patients with a clinically negative axilla, patients who have undergone breast-conserving surgery (lumpectomy) and will receive radiotherapy (especially involving the axillary area), and patients who will receive systemic adjuvant therapy (hormonal or chemotherapy) [23, 24].

In the context of SLNB, particularly for patients with early-stage tumors of smaller sizes, there is an emerging need for additional prognostic factors. TB, akin to its increasing recognition in various organ carcinomas, has been increasingly acknowledged as a significant prognostic factor in breast cancer [6]. A meta-analysis encompassing 13 studies demonstrated that high TB is significantly associated with the risk of lymph node metastasis (odds ratio 2.25; 95% confidence interval 1.52–3.34, *P* < 0.01) [16]. Our study found that the presence of TB in preoperative biopsies correlates with a higher rate of sentinel lymph node positivity.

The correlation between TB in biopsy samples and SLN positivity has significant implications for clinical practice. If TB can reliably predict nodal metastasis, surgeons may opt for more aggressive axillary evaluations, including sentinel node biopsy planning, in patients whose biopsies exhibit TB. Consequently, TB could inform decisions regarding the necessity of additional node sampling or the choice between sentinel node biopsy and full axillary dissection. Furthermore, patients identified as high-risk based on TB may require different counseling concerning prognosis and adjuvant therapy. To translate these findings into clinical practice, standardized protocols for assessing TB in biopsy specimens are essential. These protocols should define parameters such as bud size/count, the number of fields examined, cutoff thresholds, and interobserver reproducibility.

The present study has several limitations. Firstly, the sample sizes in our analysis were relatively small (36 with TB, 29 without). This limited sample size reduces the statistical power to detect differences in other parameters, such as tumor size and multicentricity, and may increase the risk of false negatives. Moreover, the method used to assess TB—specifically, the cutoff values, the number of fields examined, and the definition of buds—can significantly impact reproducibility and comparability. Secondly, methodological differences among studies can yield varying results. Standardization of TB assessment is not yet fully established in breast cancer, unlike colorectal cancer, where TB scoring is more standardized. Additionally, factors influencing both budding and nodal spread, such as tumor biology, grade, ER/PR/HER2 profile, Ki-67 index, and lymphovascular invasion, must be accounted for in multivariable analyses. Thirdly, our findings were observational and cross-sectional, suggesting that TB may serve as a marker rather than a causal driver; further elucidation of the underlying mechanisms, such as EMT, invasion, and the tumor microenvironment, is needed. While the association between TB and nodal metastasis is promising, determining whether TB in biopsy samples predicts long-term outcomes, such as disease-free survival and overall survival, necessitates prospective follow-up data. Our study was retrospective, limiting our ability to establish causality; we identified only associations. Additionally, TB was analyzed solely through tru-cut biopsy, assessed by a single pathologist, and patients with micrometastases were excluded. The lack of immunohistochemical staining to detect small-volume disease further constrained our findings. Lastly, the single-center nature of this research presents another limitation.

## Conclusion

The selection of patients for SLNB still necessitates additional prognostic factors. Current literature lacks specific studies directly examining the relationship between TB and SLN metastasis in preoperative breast biopsies, with most research focusing on postoperative histological evaluations. Therefore, incorporating TB evaluation in preoperative breast needle biopsy specimens, alongside clinical and radiological findings, may enhance the preoperative identification of patients who would benefit from SLNB.

## Data Availability

The data sets supporting the conclusions of this article and its supporting information are available from the corresponding author upon reasonable request.
